# Investigation on Identifying Implicit Learning Event from EEG Signal Using Multiscale Entropy and Artificial Bee Colony

**DOI:** 10.3390/e23050617

**Published:** 2021-05-16

**Authors:** Chayapol Chaiyanan, Keiji Iramina, Boonserm Kaewkamnerdpong

**Affiliations:** 1 Computer Engineering Department, Faculty of Engineering, King Mongkut’s University of Technology Thonburi, Bangkok 10140, Thailand; chayapol.chekl82@mail.kmutt.ac.th; 2 Graduate School of Systems Life Sciences, Kyushu University, Fukuoka 819-0395, Japan; iramina@inf.kyushu-u.ac.jp; 3 Biological Engineering Program, Faculty of Engineering, King Mongkut’s University of Technology Thonburi, Bangkok 10140, Thailand

**Keywords:** multiscale entropy, artificial bee colony, implicit learning, EEG, machine learning

## Abstract

The way people learn will play an essential role in the sustainable development of the educational system for the future. Utilizing technology in the age of information and incorporating it into how people learn can produce better learners. Implicit learning is a type of learning of the underlying rules without consciously seeking or understanding the rules; it is commonly seen in small children while learning how to speak their native language without learning grammar. This research aims to introduce a processing system that can systematically identify the relationship between implicit learning events and their Encephalogram (EEG) signal characteristics. This study converted the EEG signal from participants while performing cognitive task experiments into Multiscale Entropy (MSE) data. Using MSE data from different frequency bands and channels as features, the system explored a wide range of classifiers and observed their performance to see how they classified the features related to participants’ performance. The Artificial Bee Colony (ABC) method was used for feature selection to improve the process to make the system more efficient. The results showed that the system could correctly identify the differences between participants’ performance using MSE data and the ABC method with 95% confidence.

## 1. Introduction

There are many new and exciting technologies being invented every year. Especially now, in the age of information, the growth of new technology has grown exponentially, and there is no sign of it slowing down. To be competitive in the fast-growing world, people have to adapt and improve the learning process. Smart Education is a type of learning environment where learning can occur in a more personalized lesson plan. Utilizing available cutting-edge technology, a person can start learning more efficiently by using advanced electronics such as e-learning, online learning, hybrid learning, and blended learning to record their progress and adjust lesson plans to be more suitable to the learner. It could be a way to battle and manage all the information required to be competitive in the fast-paced technological world. Ideally, the data that would be tracked should provide feedback to the learner in real-time or as close to real-time as possible. Data, such as whether or not they are learning at that precise time, can be used to improve the learning process.

Implicit learning is a type of learning where the learning of complex information occurs in an unintentional manner. Previous research studies suggested [1] that those who can perform implicit learning well can learn other things that require similar skills more effortlessly than those who do not excel in implicit learning. Implicit learning skills are helpful for tasks such as language learning and other complex activities. Biofeedback or neurofeedback could be the tool that can be used to improve implicit learning skills.

Biofeedback or neurofeedback is a non-drug treatment technique by which individuals can self-trained or with bits of help from the therapist to improve their health through monitoring devices. The participants can learn to control bodily processes that are typically involuntary by monitoring in real-time biological and neurological data, such as heart rate, muscle stiffness, blood pressure, and concentration level. Biofeedback and neurofeedback had help patients with attention deficit hyperactivity disorder (ADHD), depression, anxiety, addiction, and other types of brain conditions [2]. There was a study that showed that 90% of children were able to improve against their ADHD symptoms, which were maintained even after a 6-month follow-up using neurofeedback training [3]. Past research suggested that one can perform better in sports activities after they undergo implicit motor learning on movement, albeit only slightly using biofeedback [4,5]. The goal of this research is to provide means to obtain data to improve implicit learning skills by introducing a concept of neurofeedback for learning events for healthy adults using the Smart Education concept. To be a better implicit learner using neurofeedback, there has to be a way to identify when the implicit learning event occurs so that we can train individuals’ brains to be better at implicit learning. The pattern of Implicit Learning events will be identified as learners go through learning activities to achieve this goal [4,5].

One way this research can discern the implicit learning event on neuro signal is to monitor said signal of the participants while they undergo an experiment where they have to solve the problem without anyone telling them how to reach the solution. We can identify when they achieve implicit learning by monitoring their brainwave, while they undergo a cognitive learning task. The measurement data that will be used for analyzing and capturing these implicit learning events are electroencephalogram data or EEG data. EEG signal is a very complex datum to interpret because they are non-linear, dynamic, and correlate between multiple channels. There is research that tried to accomplish these enormous tasks of interpreting EEG signals. Dennis et al. [6] gave a summary review of EEG-based brain–computer interface (BCI) and explored the possibility of enhancing neurorehabilitation of people with strokes and other chronic disorders. Mohammed et al. [7] introduced a new method to represent the depth of anesthesia (DOA) as compared to conventional bispectral index (BIS) monitor using wavelet–Fourier analysis (WFA_DOA_) on EEG signal.

Previously, there was an experiment on explicit memory during learning conducted by Rose et al. [8]. The experiment was set up where 31 participants were asked to solve a cognitive problem without any prior instruction on how to solve that problem. Each of these cognitive problems will be consist of four questions per trial and up to 180 trials per participant. There will be two rules that govern all the questions in the experiment. The 4th question will be different in that there will be one more additional rule to govern it. Using the response time of the 4th question and compare it to the response time of the average of the other question, there is a set of algorithms that will determine whether learning had occurred or not. Triple Response Time (TRT) was coined up by Rose et al., which is when the response time of the 4th question was consistently faster than the response time of the average of the other question in the same trial for three consecutive trials [8]. During the Response Time Analysis phase of our research, we discovered that TRT had occurred in both of our groups between those we determined to have achieved implicit learning and those that did not. It was decided that TRT alone will not be enough to be used as a marker, but it’s a start. Entropy was selected after many literature reviews to be used alongside TRT to differentiate between the two groups. Markers such as these can be used to help our research narrow down when the implicit learning event occurs.

Given that the raw EEG data are inherently challenging to discern, it is imperative to modify and convert the EEG data into more manageable elements. There are studies that use EEG entropy data as a way to identify different brain states [9]. Entropy is a scientific concept for the measurement of uncertainty and complexity within a system. By converting brain signals into entropy data, researchers would be able to measure the level of complexity in the brain, and in turn, able to determine the activeness of the brain state. Since brain signal is always complex, the level of complexity can differentiate between brain states. Case in point, when a patient entropy value is of a certain level, it means the anesthetic is working, and the brain is at a more relaxed state [10]. Entropy value has also been shown to be able to differentiate between memory and motivation. The difference between wanting to remember or not and actually remembering or not can have different entropy values for the duration of the event [11].

While entropy is useful in finding the brain state based on fixed-time events, it is not as useful to find a pattern in time series data. This is because the event pattern itself is often not known, not even the duration of the event. There are studies that used Multiscale Entropy (MSE) and see its scaling effect on the data to identify patients with heart failure with reliable success [12]. The multiscale entropy measure differs from regular entropy techniques in that it included multiple time scales of measurement using a coarse-graining method. The inclusion of these multiple measurements allows for assessing complexity in an overall individual time scale and at longer and shorter time scales. By taking all time scales into account, this combination of features allows researchers to identify the time scale at which the peak in complexity occurs. The overall time scales or scaling effect of the data at the preset period of time will be the feature that will be analyzed to find whether of implicit learning event had occurred or not due to its flexibility and robustness in capturing the level of complexity in an unknown data pattern. Donglin et al. [13] proposed a novel multi-scale fusion convolutional neural network based on an attention mechanism for the visualization analysis to improve signal representation, the robustness of the network system, and maximize information flow. Wonjun et al. [14] improved upon classifying motor imagery (MI) induced brain signal by differentiating between relevant and irrelevant information using the reinforcement learning method and discarding the latter. Wonjun et al. [15] also introduced a novel deep multiscale neural network (MSNN) to extract information among spatial representations for subject intention and condition identification. More precisely, they exploited multiple EEG paradigms in the network contrary to the prevailing methods of focusing on one EEG paradigm to classify early seizure detection and mental fatigue.

There were several discussions and research in regard to using recurrent neural network (RNN) methods such as long short-term memory that seem to work well with time-series data or deep learning-based BCI methods such as convolutional neural network (CNN) to deal with EEG classification of brain state at various application [16]. These methods do not seem to work well with our approach. One major reason is that our data is simply not large enough for these data-hungry methods to yield a good result. According to Ovedare studies on data size and their corresponding classification performance using deep learning, our data size is simply too small to be used in CNN and LSTM effectively [17].

The objective of this study is to systematically identify the relationship between implicit learning events and their EEG signal characteristics by searching for distinguishable features that related to participants’ performance and test the efficacy of different classifiers to find those differences. This study will explore a wide range of classifiers and their performance at correctly classifying the EEG signals related to participants’ performance. Our contribution is simply that this paper will introduce a method that will identify the implicit learning event using MSE based feature extraction approach for event detection in time series. This paper will evaluate whether our proposed method of using MSE in conjunction with artificial bee colony can deliver a satisfying outcome with statistical confidence in high accuracy.

## 2. Data Collection

Implicit learning is commonly found in small children learning how to speak their native language without prior knowledge of grammar. Past research showed that many who succeeded in implicit learning could perform other tasks that require similar skills, such as learning an additional language [1]. The data used in this research was acquired from an earlier experiment conducted by our previous experiment. The experiment was designed to captured implicit learning events. Subjects will be consisting of 30 people between the age of 21 and 29 years old. They will be healthy with no prior learning disability and no color-blindness. Throughout the entire experiment, the participant will be strapped to an EEG and fNIRS cap, while they undergo visual event-related potentials (vERP). All participants were volunteer that signed written informed consent, and the experiment was approved by Experimental Ethics Committee of Faculty of Information Science and Electrical Engineering, Kyushu University (ISEE H26-3, 23 June 2014).

**Figure 1:**
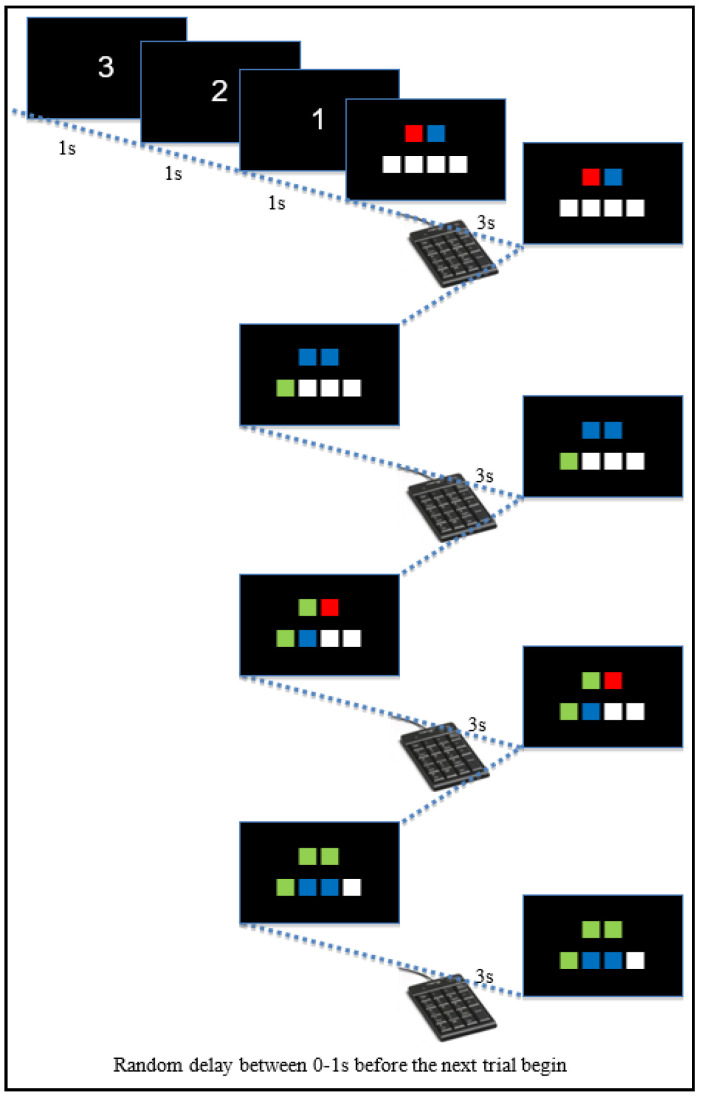
Cognitive tasks designed to induced learning.

**Figure 2:**
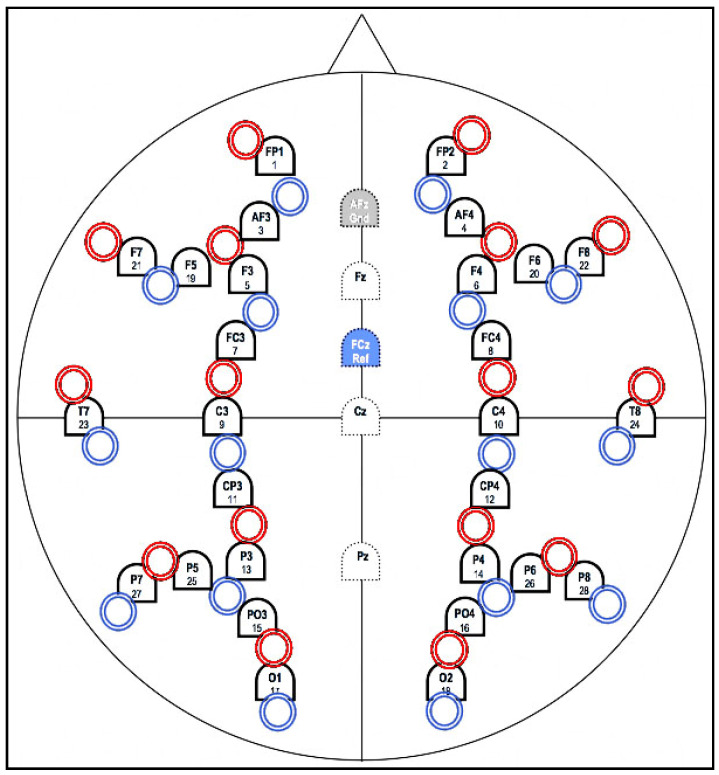
10/20 system positioning was used to record EEG/NIRS. Red and blue circles represent the emitter and receiver of NIRS optodes, respectively, while the arches symbols represent the EEG electrodes. Noted, NIRS data will not be discussed in this paper.

The experiment will be consisted of two boxes on the screen and requested to respond based on what they see on the screen by pressing the color buttons on the keypad with no prior instruction. Each of the boxes will have its corresponding color. The participant was asked to respond within three seconds. There will be a total of three different colors which are red, blue, and green. The participants were asked to input one of those colors in response to the paired color shown on the screen. Once the 3 s are up, the correct answer will be shown alongside the following pairs of color boxes. These answers will follow a particular set of rules which is not known to participants. Each trial will consist of four questions. There will be a random delay between one to zero seconds in between each trial. There will be a maximum of 180 trials per participant. If the subject seems to grasp the underlying rules before the 180 trials were up based on the observer’s discretion, the experiment will continue for ten more trials then stop. The experiment is shown in Figure 1 and Figure 2.

The answer to the color pair questions will be governed by three rules. The first rule stated that if the color pair is different in color, the answer will be the color that is not shown. If the color pair is the same color, the answer will be that color; that is the second rule. When the participant input an incorrect answer, there will be a beeping noise to indicate negative feedback that the answer was incorrect. The last rule state that the answer to the first color pair will always be the answer to the fourth color pair question. Please note that there is no indication or feedback of any kind to hint to the participant of this rule, such as a feedback sound like for the first two rules.

Whether or not the participant achieves the status of explicit and implicit knowledge was evaluated immediately after the session had been interrupted or concluded. The participants were asked whether they noticed anything special about the task. If a participant were to answer yes, they were asked to explain. If they did not notice anything special, then it would be explained to them that there were rules governing the tasks. They are then asked to describe and articulate the rule and to write down examples. If the participants were able to describe the first two rules, then they will be categorized as having an explicit learning event. If the participant could verbalize the 3rd rule, that participant was then considered as a group that has obtained implicit learning during the experiment. This information is crucial because it will be used for supervised learning in the later process of the research.

Notice that our experimental design was similar to Rose et al. [8]. The difference between Rose et al.’s and our experiment is how there were negative feedbacks provided to the participant in the form of beep noise when they provide an incorrect answer. This feedback is important because it provides crucial information to the participant’s ability to answer the question. However, this negative feedback does not represent the 3rd rule that governs only the 4th question. The rule stated that the answer to the 1st question will always be the same as the answer to the 4th question. These differences between the 1st, 2nd, and 3rd question and the 4th question will have an impact on how participant response time (RT). It will be the crucial distinction of when implicit learning has occurred or whether they happen at all.

## 3. Methods

**Figure 3:**
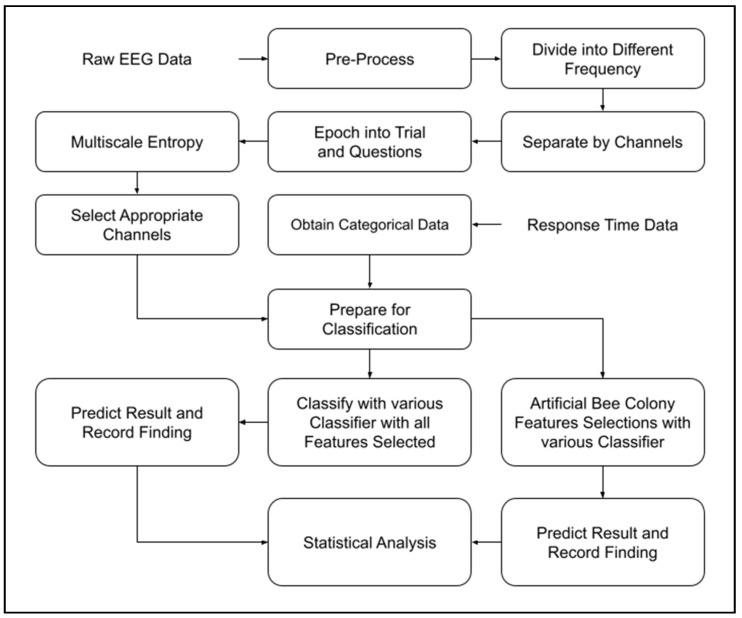
Analytic Process of the EEG and Response Time Signal that was done for this paper.

In this study, Multiscale Entropy was used as a method for feature extractions, while Artificial Bee Colony (ABC) algorithm was used for feature selections. An examination of various machine learning techniques will be applied to see how effective ABC methods are on the current setup according to the block diagram, shown in Figure 3.

### 3.1. Entropy and Multiscale Entropy (MSE)

The concept of entropy was first used by Rudolph Clausius in the field of thermodynamics [18]. It basically follows the second law of thermodynamics that defined the change of the entropy in the volume elements as equivalent to the ratio between changes in heat state and temperature [19]. Since then, “Entropy” was used as a measurement of the system’s thermal energy per unit temperature or the molecular disorder, in turn, the randomness of the system. Using that concept of the randomness of the system, Shannon [20] started using entropy to define the amount of complexity or useful information of a system in the field of information theory, the field he created. Right now, there are various usages of entropy and its evolution in the field of biomedical signals [21,22,23]. Pincus [24] used approximate entropy to examine the changes in the heart rate of infants to help identify the sudden illness. Approximate entropy was determined to be well suited to solve the problem of common signals with short noise in the biomedical signals. Richman and Moorman further developed sample entropy (SampEn) which works better on data with varying data lengths which is very suitable for biomedical data [25,26].

The humans’ vital signs, such as EEG signals, are quite erratic and prone to noise interference. A signal conversion must be implemented to observe and determine its behaviors correctly. Entropy, a concept that represents the complexity of information, when applied to bioinformatics data can simplify the erratic signal and turn it into a more manageable representation. The value of the entropy will increase or decrease based on the level of complexity of the EEG signals. If the entropy value is relatively low for a certain region of the brain, it means the brain is less active in that particular region. Sample Entropy was chosen in this research due to its robustness and capability to adjust for real-time detection [10].

Converting the raw EEG data into SampEn data, the EEG signal must first be divided into short sequences, called epochs. Using the entirety of that epoch data to determine the SampEn by applying it to:

(1) $$\mathrm{SampEn}\left(m,r,N\right)=-\log\frac{\sum A_{i}}{\sum B_{i}}=-\log\frac{A}{B}$$

where m is the template length, r is the tolerance for accepting matches, N is the number of data points per epoch, A_i_ is the number of the matches of length m+1, and B_i_ is the number of matches of length m. If the number of matches is equal for A and B, the SampEn is equal to zero, or no complexity in the data. If the number of matches for template A is smaller, and it will always be the same or smaller because the template is shorter, then the SampEn value will increase.

While SampEn is acceptable for determining the complexity of the data in fixed length, the determination of that length is still up for contention. Which is how Multiscale Entropy (MSE) was introduced. Determining MSE, the epoched data will be coarse-grained by averaging in the range of a specified scale. If the scale were 2, two neighboring data points are averaged. The number of total data points is reduced to a total number divided by two. The new dataset can be expressed through the scaling process as [27,28,29]:

(2) $$y_{j}^{\left(\tau \right)}=\frac{1}{\tau }\sum_{i}=j\tau -\tau +1^{j\tau }x_{i},1\leq j\leq \frac{N}{\tau }$$

where x_i_ is the original data point, and τ is the scale level, then the sample entropy will be calculated from the new dataset for each scale.

Previous studies have shown that SampEn’s most appropriate template length m to be 2, r = 0.15x standard deviation of the epoch [27,29] for time series data. According to Richman and Moorman [25], the appropriate N should be at least 100 to 400 for each scale because when the scale increase, the number of N will decrease. Based on the experiment data that was gathered that would be explained in the later section, the sampling frequency is 1000 Hz, with the appropriate epoch for the experiment to be at maximum, 3 s, we determined that our data will have at most 1000 Hz × 3s = 3000 data points. With the maximum scale of 20, the size of N is 150, which is still enough data points to get a reliable SampEn.

### 3.2. Artificial Bee Colony

Artificial Bee Colony (ABC) [30] is an empirical method that tries to mimic the intelligent action of honeybees while they hunt for their food source. Although ABC is very similar to Ant Colony Optimization and Particle Swarm Optimization as they share information between members in the colony, ABC is easier to implement because it has a smaller number of control parameters to adjust. In addition, ABC also has a unique solution update process that allows the result not to get stuck in a less optimal solution and instead converge on the actual optimal solution. Bee colonies tend to operate by dividing their duties and sharing information about food sources among other bees in their perspective colony. Bees in the ABC algorithm are divided into three types: employed bees, onlooker bees, and scout bees. Employed bees evaluate current food sources and share the information with onlooker bees that reside in the hive. With that information, the onlooker bees will decide on whether they will go out and find the food source or not based on the received information. Scout bees are tasked with the search for new food sources. The determination of which food source they will utilize will be decided based on the number of food sources and their quality. In ABC, a food source represents a solution to an optimization problem. Fitness function will determine food sources’ quality at a given location. This behavior of bees searching for a source of food can be utilized to locate the optimal solution in the given system.

ABC can be divided into three steps that will be looped and repeated accordingly. After initializations of the food sources, which are randomly created to within a predetermined range, the following formula will be used for the first step, Employed Bees Phase:

(3) $$v_{ij}=x_{ij}+\mathrm{rand}\left(0-1\right)\left(x_{ij}-x_{kj}\right)$$

the subscript *k* and *i* indicate the solution number, and the index *j* indicates the dimension vector of each solution, that is, the location of the solution in the space of all solutions. Using the current solution *x_ij_* and a randomly selected neighbor *x_kj_*, *v_ij_* was created. Rand function was used as a uniformly distributed random number generator between zero and one. This function will randomly generate a new solution in the next iteration from the current solution. The employed bees and onlooker bees will replace their current position with the new solution if the new candidate solution has a better fitness value than the residing one.

The second phase, the onlooker phase, is when the onlooker bees will use the known information from employed bees to determine the possible solution with a probability that depends on the relative fitness of each solution based on this fitness equation:

(4) $$P_{i}=\frac{{\mathrm{fit}}_{i}}{\sum_{n}^{N}{\mathrm{fit}}_{n}}$$

P_i_ was calculated for the probability of each I solution. N is the total number of all solutions, and fit_i_ represented the fitness value of each solution. Fit_i_ was generated from the fitness function, which is based on the objective function of the problem. As the fitness value increases, the probability that the new solution will be updated by the onlooker bees will also increase. The objective function that will be used in this research will be the accuracy of the classifiers.

The third phase, the scouting phase, is used to avoid suboptimal solutions. The scout bee process will start when an employed bee is stuck in the same location over a preset number of trials. This can happen when an employed bee cannot improve its fitness value over and over again. If this were to occur, the current solution would be abandoned. The employed bee will turn into a scout bee and start searching for new solutions.

## 4. Analytical Process

Our research will flow as shown in Figure 3.

### 4.1. Pre-Processing Data

Bandpass filtered of 0.5–50 Hz and a Notch filter of 60 Hz was used on the EEG data to remove physiological and power line noise, respectively. Furthermore, a blind source separation method (BSS) called independent component analysis (ICA) was used to remove more of the non-brain signal [31].

### 4.2. Feature Extraction Using Multiscale Entropy

**Figure 4:**
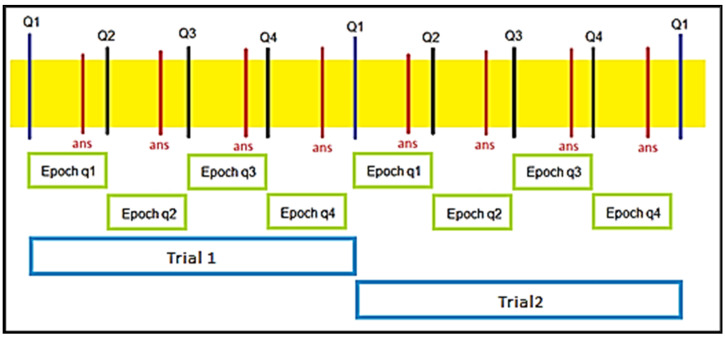
Sample Entropy data are divided into the epoch. Each epoch consists of the duration from when the question appears until the beginning of the next question.

When computing for SampEn, the size of what to be calculated must also be considered. Since the data consists of many trials and they are all in a time series, dividing the data into appropriate epoch must happen first before SampEn data get implemented. There are many things to consider; since we have different participants and each participant has a different number of trials. Moreover, based on the setup of the experiment, it is also known that in the trials, there are four questions, and only the 4th question got dictated by the extra rule; therefore, it is essential to separate the sample entropy calculation into whether implicit learning had occurred or not. As shown in Figure 4, we perform Multiscale Sample Entropy on each epoch, where an epoch is between the time question is asked and when the next question will begin. This means that each trial will produce four multiscale sample entropy data.

### 4.3. Obtain Categorical Data and Response Time Analysis

According to the Response Time Analysis and SampEn data, there is a trend that seems to indicate the difference between the group of those who achieved implicit learning and those that did not. One of those trends is that the group with implicit learning tends to achieve Triple Response Time (TRT) more regularly as well as maintaining that state for a longer amount of trials compared to the group that did not. TRT was decided to be event marker based on that distinction.

Obtaining the TRT label, the response time data were divided and modified into two types. The moving median (MM) of window five was performed on all response time data to get rid of any outlier. The MM response time for 4th question was categorized as determined response time (DRT). The average MM response time between 2nd question and 3rd question was performed for each trial and given the label of undetermined response time (URT). When DRT drops below 90% negative confident interval of URT for three consecutive trials, that is when the mark of TRT was given to that trial. In total, we were able to extract 175 trials where TRT was achieved from those we determined to have achieved implicit learning. We also randomly selected 175 trials from the group that we determined as people who did not achieve implicit learning during the experiments to have an equal number of data for a non-bias classification process.

**Figure 5:**
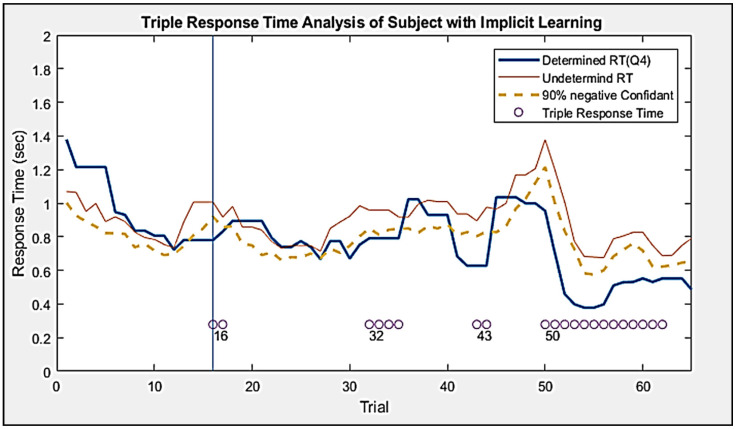
An example of the Response Time Data. Determined RT is the response time of Q4, while the Undetermined RT represents the average response time of 2nd and 3rd question in the same trial. The dotted line is the 90% negative confident interval of the Undetermined RT. Each dot represented a trial that was given the fast response time label. Fast is the response time of Determined RT that is less than the response time of the Undetermined RT negative confident interval for three consecutive trials.

The epoch in which they achieved TRT were given fast labels, while the epoch that did not achieve TRT received slow labels. These labels were used to train various classifiers as supervised learning. Figure 5 showed a graphical representation of the TRT marker.

If the participant was able to perform significantly better at answering 4th question as opposed to the other question, it must mean that a learning event had occurred at some point, either at this particular trial or some trial earlier. This means that the trial that received TRT marks are very crucial at identifying the learning event.

### 4.4. Preparing Features Selections

The feature selection process is a process that will identify important features and remove redundant or irrelevant features from the entire set of features. Unlike feature extraction, which tends to use all features in its classifier, the feature selection method will search for and identify the optimal subset of feature data from all the available features that will still yield adequate results while maintaining the least amount of error and information loss [32].

The features to be selected are the frequency band and the EEG channels. In preparing for classification, only the frontal channel of AF3, AF4, F3, F4, F5, F6, F7, F8, and FZ, were chosen. These channels were selected because the frontal lobe was identified as a region that is responsible for personality expression, planning complex cognitive behavior, memory, and decision making [33]. These channels and frequency bands, including Gamma, Beta, Alpha, Delta, and Theta, will be assigned as features. If the features were to be selected, the feature would be represented as 1. If the feature is not selected, it will be represented as 0. These strings of zero and one will be Food Source for the ABC [34].

### 4.5. ABC Process

**Figure 6:**
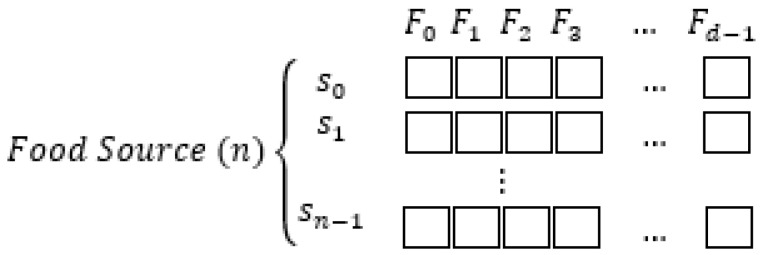
Food Source for ABC process. *S_n_* is the bee, and *F_d_* are features.

This section will describe the setup and methods for the ABC algorithm as seen in Figure 6.

#### 4.5.1. Initialization

To begins, several parameters are needed to be considered. The number of employed bees, onlooker bees, the threshold for scout bees to be deployed, and the maximum number of iterations are the parameters that needed to be decided. Once that is done, the initial food sources are generated randomly as well as their respective solution. The solution is derived from the various classifier. The accuracy value from each classifier will be used to represent the food source fitness value. Simply put, the higher the fitness value, the better the classification performance.

#### 4.5.2. Employed Bees Process

Employed bees process is a process where food source gets updated based on the solution of the prospective food source location expressed in Equation (5). It was decided that in this algorithm, the food source will be binary numbers. It follows the concept of the solution update based on the nearby solution using the bitwise AND operation as follows:

(5) $$v_{ij}=\left\{\begin{array}{cc} x_{ij}=x_{kj} & if\left(x_{ij}=x_{kj}\right)\\ 0 & if\left(x_{ij}\neq x_{kj}\;\mathrm{and}\;\varphi \;\leq 0.5\right)\\ 1 & if(x_{ij}\neq x_{kj}\;\mathrm{and}\;\varphi >0.5) \end{array}\right.$$

where *v_ij_* the new candidate for each food source location *i* and *j*, *x_ij_* the current location of the food source *i* feature *j*, *x_kj_* the randomly selected neighboring location *k* feature *j*. *F_d_* represented in Figure 4 are features that will be selected or not based on a random real number between 0 to 1, where *d* is the total number of potential features.

In this method, the feature value will be either 0 or 1, for not selected and for selected, respectively. If the value of the feature j given from the neighboring food source and its present food source are the same, then there will be no changes to the current food source location. Otherwise, a new value of either 0 or 1 will be randomly assigned as a new food source location. For example, if a current solution is {0, 1, 1, 0}, and the selected neighboring solution is {0, 1, 0, 1}, the new candidate solution could be one of the following solutions {0, 1, 1, 1}, {0, 1, 1, 0}, {0, 1, 0, 1}, and {0, 1, 0, 0}. Once the new location is obtained, the new solution will be generated using the various classifier. If the new solution and its fitness value were deemed to be better than the current one, the employed bees would then update their solution with the new candidate solution. If the new solution is not better, then the solution will be ignored.

#### 4.5.3. Onlooker Bees Process

Onlooker bees will select whether to visit a new food source once the employed bees share their information of their solutions based on the probability of each respective food source, based on Equation (4). Updating possible solutions using the same methods as employed bee phase using Equation (5) will also be performed in the process. If the new solution has a better fitness value, the current solution in the onlooker bee’s memory will be swap by the new candidate solution.

#### 4.5.4. Scout Bees Process

The current food source location will be abandoned if the fitness value of the current food source has not been improved by the decided number of iterations, called “limit.” The scout bee will then randomly create a new food source location in the system space in all dimensions if the abandon counter (AC) is above the limit. This way, a sub-optimal solution will be avoided. The Best So Far of the food source location, however, will not be forgotten as it is stored in best so far solution trackers.

#### 4.5.5. Termination Process

The entire operation will be repeated until a maximum number of iterations is reached.

### 4.6. Classifier

Evaluating the solution of the food source location, the various classifier will be used and examine its performances. The classifiers are Decision Tree, Random Forest, kNN, and SVM. These classifiers were chosen to be surveyed because to properly identify implicit learning events to as close to real-time as possible with accurate results; many factors need to be considered. Factors include accuracy, robustness, computer memory, and CPU usage, interpretability, and speed. Knowing the characteristics of these classifiers will be helpful for future research on whether to evaluate learners in real-time or posthoc analysis.

#### 4.6.1. Decision Tree

A decision tree is a type of classifier where it will generate a flowchart-like structure from each node that represents an attribute of each branch. Each leaf node is a class label within each branch which in turn represents the test condition. When putting it all together, the paths from leaf to root will stand for classification rules, equations, or conditions for each class label. The number of maximum nodes will be set to 100, 20, and 3 for Fine, Medium, and Coarse configuration, respectively, of decision trees that were evaluated in this paper.

#### 4.6.2. Random Forest

Random forests (RF) are an ensemble of a large number of individual decision trees. The final prediction will be derived from the predictions from all trees that were pooled together. Each decision tree in the random forest will generate its own class prediction. The class with the most predictions will become the classifier’s prediction.

#### 4.6.3. k-Nearest-Neighbor (kNN)

k-Nearest Neighbor is a non-parametric, lazy learning algorithm. It stores all instances that correspond to training data points in n-dimensional space. Its purpose is to use data and its corresponding classes to predict the classification of a new sample point. Once a new unknown discrete data is received, it analyzes the closest possible class it should belong to base on the number of class membership and returns the most common class as the prediction. There will be four configurations of kNN classifiers that were used in this paper. Three kNN will use Minkowski Distance with a number of neighbors being 1, 3, and 20 for Fine, Medium, and Coarse, respectively. The last kNN configuration that was evaluated will use Cosine Distance.

#### 4.6.4. Support Vector Machine (SVM)

Support vector machines are algorithms used to find a hyperplane in an N-dimensional space that distinctly classifies the data points. N is often representing the number of features. The hyperplane that was chosen will determine the accuracy result of the model. The most optimum plane should have the maximum distance between data points of different classes. There will again be four configurations of SVM classifiers that were used in this paper, each with different kernel functions. The three polynomial kernel functions that were used were linear, poly, and cubic. One SVM used Gaussian RBF as its kernel function.

### 4.7. Statistical Analysis

Once feature selection was completed, using the best food source location, a new classification evaluation will be performed five times for each location. The results were evaluated using *k*-fold cross-validation. Here, the *k* value was chosen as 5. Each result will also yield a confusion matrix which will be further used as a performance metric to determine accuracy, specificity, and sensitivity. An independent-samples *t*-test was performed to compare the difference between using the ABC method and not using the ABC method for classification on each classifier. This will tell us which classifier provides the best accuracy as well as which features were selected. This *t*-test will also tell us that the difference between using ABC and not using ABC will be significant or not.

## 5. Results and Discussion

In this section, we present our ABC results in identifying the differences in participant’s performances while they undergo their cognitive tasks.

**Figure 7:**
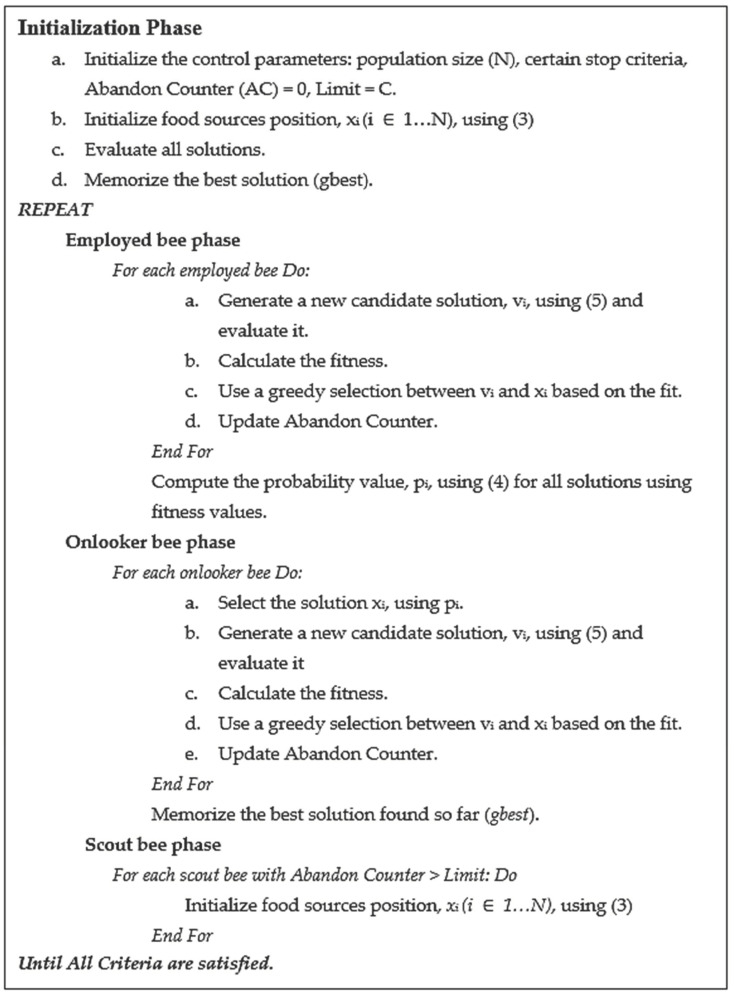
Pseudo code of the ABC algorithm.

**Figure 8:**
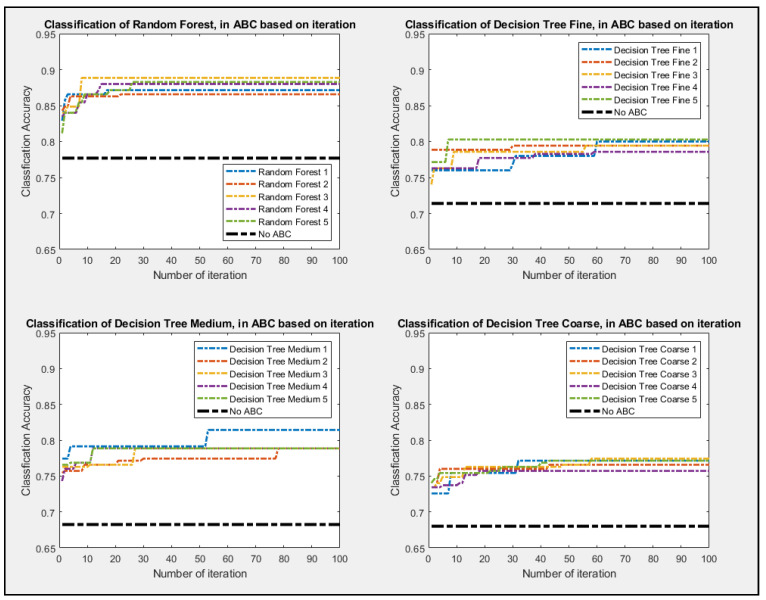
Best So Far Accuracy value as ABC performed its feature selections for Random Forest and three set up of Decision Tree Classifier.

**Figure 9:**
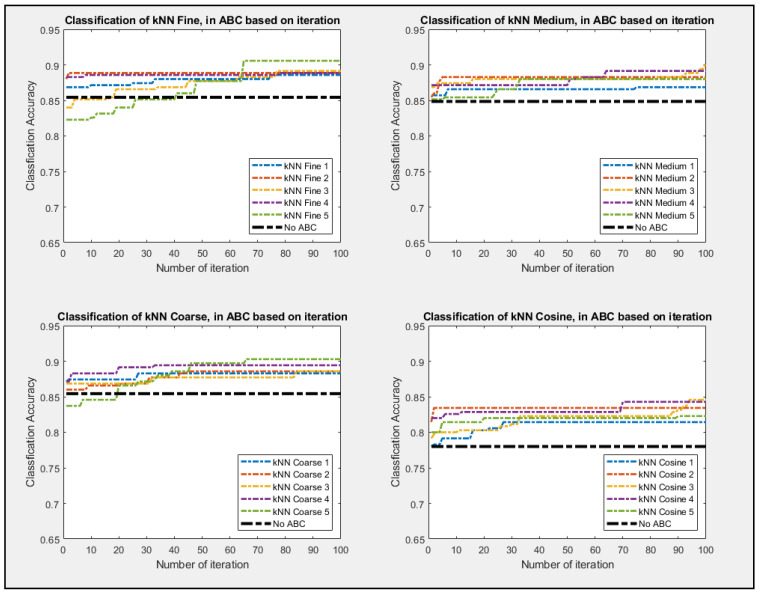
Best So Far Accuracy value as ABC being performed of the four setups of kNN.

**Figure 10:**
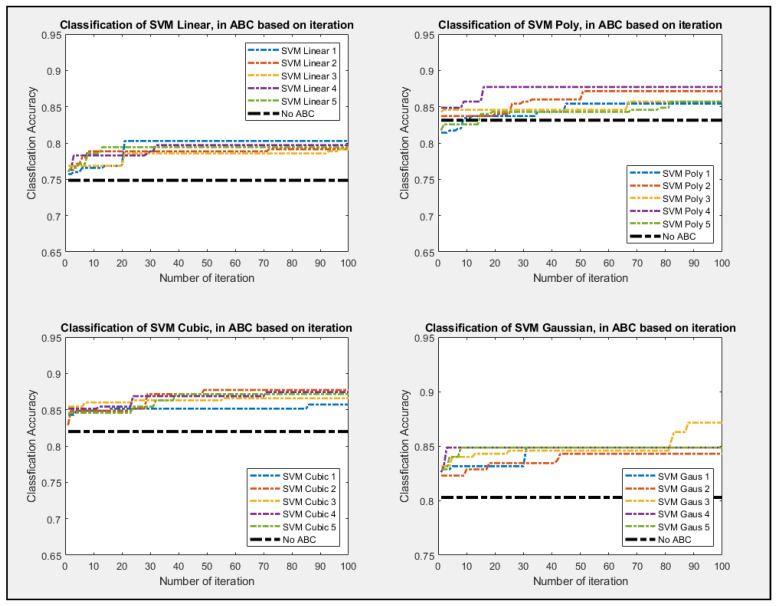
Best So Far Accuracy value as ABC performed of the four setups of SVM.

Given how classifier are inherently governed by probability, the experiment was repeated five times for the ABC feature selection process. The non-ABC process was also conducted with all features being selected while it went into the classifiers. The non-ABC was again repeated five times, and only the median value was represented as in Figure 7, Figure 8 and Figure 9 as a thick dot-dash black line. Since non-ABC has no feature selection, the accuracy of the classifier will not change as iteration increases. As shown in Figure 8, all four setups of the classifiers perform better as iteration increases. The four set up of the decision tree classifier for Fine, Medium, and Coarse are tree split of 100, 20, and 3, respectively. All of the ABC processes consistently outperformed their non-ABC counterpart. Figure 9 showed that kNN classifiers and all of their configurations pretty much outperform all the other classifiers as well. The configuration for the kNN classifier for Fine, Medium, and Coarse are the number of different neighbors of 1, 3, and 20, respectively. Given that kNN tends to be much slower and very taxing to memory usage, it might not be advisable to use this classifier for real-time analysis, even if its performance outshines the other classifier. Figure 10 showed the ABC process using SVM classifiers and all of its configurations. The configuration for the SVM classifier for Linear, Polynomial, Cubic, with the degree being 1, 2, and 3, respectively. SVM classifier also used Fine Gaussian as the last configuration.

The ROC curves showed a clear distinction between how the classifier performs better with feature selections, as shown in Figure 10. Given how generating the ROC curve requires randomly partitioning of the data with fivefold cross-validation, the individual classifier performed the cross-validation with the selected best food source location five times. The ROC that is represented as the result of the median of those cross-validations process to make sure that the result is fair.

**Figure 11:**
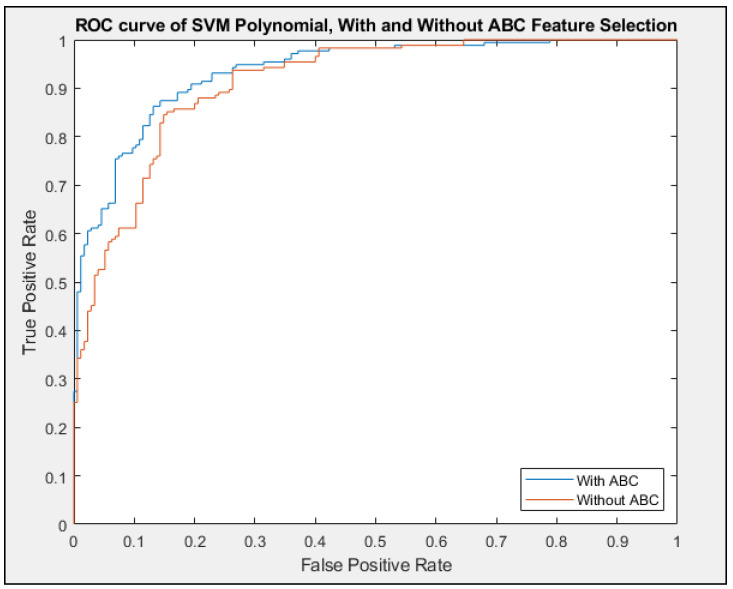
ROC curve between the Without ABC and With ABC at the end of the feature selections. SVM Polynomial was chosen as a demonstration for the ROC curve at random.

**Figure 12:**
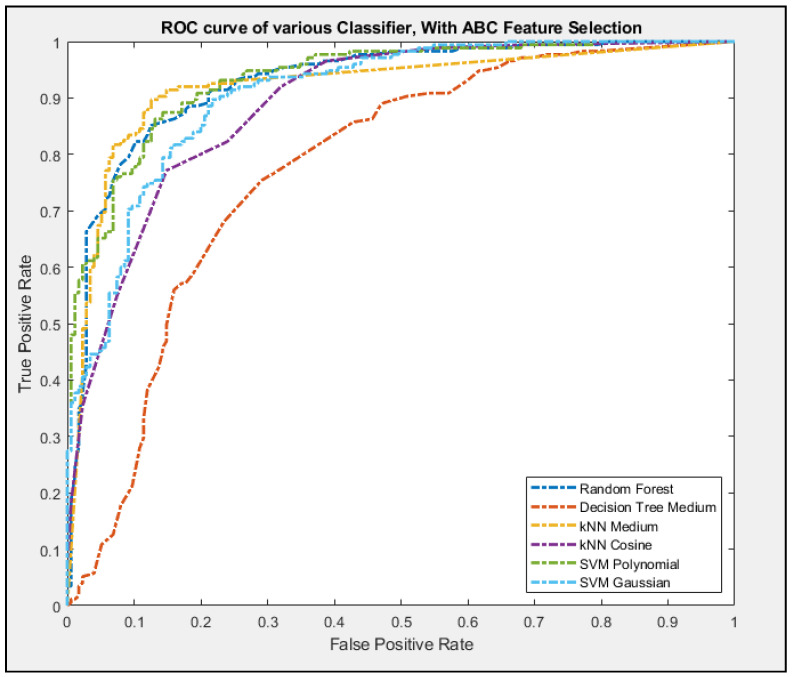
The ROC curves of each of the classifier with one configuration for each classifier.

Using the same cross-validation process as in Figure 11, Figure 12 showed the ROC curves for all the classifiers. Only some configurations of the classifier were chosen as not to clutter up the graph. Based on the data, it is clear that Decision Tree performed the worse while kNN performed the best in terms of getting the most accurate results.

Once the feature was selected, the best food source location will be used to reevaluate the classifiers for accuracy, specificity, and sensibility. Each food source will be reevaluated a total of five times, and the median value will be recorded as presented in Table 1 and Table 2. Table 1 represented the classifiers’ accuracy, specificity, and sensibility as well, but all features will be selected as they never went through the ABC feature selections process while Table 2 represented the confusion matrix outcome of ABC method. Table 3 and Table 4 showed the best, median, mean, and standard deviation of accuracy values for each classifier without using ABC method and with using ABC method respectively.

*t*-Test was conducted to see whether utilizing ABC has a significant difference compared to not using the ABC method. The null hypothesis was set to that they are the same. Table 5 showed that the accuracy from 11 out of 12 classifiers has a *p*-value less than 0.05; therefore, of those classifiers, the Null hypothesis was rejected. Also, of those classifiers, their respective Median value is shown to have a positive difference, so a conclusion can be formed that using the ABC feature selection process will yield better accuracy result with 95% confidence. As for Specificity and Sensitivity, 6 out of 12 classifiers and 9 out of 12 classifiers have *p*-value less than 0.05, respectively, which means that the system tends to be able to classify fast events correctly compared to classifying slow events.

We also performed a performance test of our system to see whether it can identify the fast trial not used in the trained data. Our data with a fast label was very limited to begin with, but nine data that were leftover was used to perform this evaluation. To make a fair comparison, we also obtained nine non-train slow trials and saw if the system can identify those trials as well. Our confusion matrix result is shown in Table 6. We randomly selected one set of parameters for each classifier because the result does not change much from one configuration to another. Our system was able to classify the non-train test trial with accuracy ranging from 67% to 94% for the system not utilizing ABC and 83% to 100% when utilizing the ABC method. These high accuracy performances are skeptical, due to very small test data, but at least we can ascertain that our system was able to identify fast and slow trials outside of the trained data with fewer features while still yielding relatively high accuracy.

In summary, our system was able to confidently identify the fast response time from the slow response time based on MSE data. This means that our system has the potential to identify fast and slow trials, which can be used in the future to identify implicit learning events.

## 6. Conclusions

In this study, a design scheme of a way to capture features of the implicit learning event and its EEG signal characteristics was introduced. Many EEG data transformation was performed to accomplish this. First, the data was converted to Multiscale Entropy data to simplify the data for features extraction. The features are then marked by event markers TRT, captured from Response Time Analysis, to be used to supervise learning of whether the participant has some kind of learning or not at that point in time. Features are then classified by many classifiers. The result is the baseline of what the research was trying to improve. Features selections methods were implemented to increase the performance for capturing the features of the learning event. The experimental results showed that the ABC method indeed improved the performance of features extraction.

Although the features extraction and selections process proposed in this paper showed that learning events can be identified with high accuracy, there can still be a further improvement on the following aspects: (1) The ABC that was used was not dynamic. There are still ways to improve upon this ABC process to cater specifically to our binary data type of feature selection. (2) The spatial location and the frequency band can still be analyzed to narrow down feature selections to speed up the process and make it more efficient. Based on preliminary observation, our results do coincide with the finding from Rose et al. in terms of spatial location and frequency band. This finding could play an important in identifying learning events. (3) This is still very early to accomplish the overall goal of finding the implicit learning event. Many things are still needed to be accomplished. For example, identifying the trial where the participant’s performance was improved does not necessarily mean that the learning event had occurred in that trial. More than likely, the learning event should have occurred a trial or two before the TRT trial. Identifying the TRT means that the participant started to use the knowledge to improve his relative response time.

By continuously improve on the brain–computer interface technology at identifying mental activity associated with the learning process and how to apply it in real-time, a system based on BCI can provide an adaptive learning environment which can enhance the learning process that can be utilized in a Smart Education environment. A marriage between BCI and education can and will provide a better learning process for all members of society.

## Figures and Tables

**Table 1:** Classifiers’ performance when all available features were selected.

Without ABC										
	**Random 1**	**Random 2**	**Random 3**	**Random 4**	**Random 5**	**Tree Fine 1**	**Tree Fine 2**	**Tree Fine 3**	**Tree Fine 4**	**Tree Fine 5**
**Accuracy**	0.791	0.762	0.777	0.78	0.76	0.6914	0.72	0.6912	0.714	0.7229
**Specificity**	0.7714	0.7771	0.7486	0.791	0.7143	0.7286	0.6514	0.7143	0.6943	0.6971
**Sensitivity**	0.7943	0.7314	0.7371	0.8	0.8057	0.6971	0.68	0.7886	0.6971	0.7486
	**Tree Med 1**	**Tree Med 2**	**Tree Med 3**	**Tree Med 4**	**Tree Med 5**	**Tree Coarse 1**	**Tree Coarse 2**	**Tree Coarse 3**	**Tree Coarse 4**	**Tree Coarse 5**
**Accuracy**	0.68	0.682	0.6824	0.72	0.7171	0.7	0.66	0.68	0.69	0.6457
**Specificity**	0.7286	0.7543	0.7429	0.72	0.7143	0.7714	0.76	0.6686	0.7143	0.6571
**Sensitivity**	0.7057	0.7029	0.6457	0.7086	0.72	0.7486	0.76	0.6457	0.7257	0.6343
	**kNN Fine 1**	**kNN Fine 2**	**kNN Fine 3**	**kNN Fine 4**	**kNN Fine 5**	**kNN Med 1**	**kNN Med 2**	**kNN Med 3**	**kNN Med 4**	**kNN Med 5**
**Accuracy**	0.84	0.8543	0.8314	0.8314	0.84	0.8486	0.8343	0.8571	0.8429	0.8543
**Specificity**	0.9429	0.9486	0.9257	0.9314	0.9429	0.9486	0.9429	0.9543	0.96	0.9543
**Sensitivity**	0.7371	0.76	0.7371	0.7314	0.7371	0.7486	0.7257	0.76	0.7257	0.7543
	**kNN Coarse 1**	**kNN Coarse 2**	**kNN Coarse 3**	**kNN Coarse 4**	**kNN Coarse 5**	**kNN Cosine 1**	**kNN Cosine 2**	**kNN Cosine 3**	**kNN Cosine 4**	**kNN Cosine 5**
**Accuracy**	0.8543	0.8657	0.8429	0.8486	0.8657	0.7771	0.7886	0.76	0.7829	0.78
**Specificity**	0.9486	0.9429	0.9486	0.9714	0.9486	0.8229	0.8457	0.8171	0.8514	0.7943
**Sensitivity**	0.76	0.7886	0.7371	0.7257	0.7829	0.7314	0.7314	0.7029	0.7143	0.7657
	**SVM Linear 1**	**SVM Linear 2**	**SVM Linear 3**	**SVM Linear 4**	**SVM Linear 5**	**SVM Poly 1**	**SVM Poly 2**	**SVM Poly 3**	**SVM Poly 4**	**SVM Poly 5**
**Accuracy**	0.7486	0.7571	0.7543	0.74	0.7257	0.8429	0.8314	0.8257	0.8114	0.8371
**Specificity**	0.76	0.7429	0.7371	0.7086	0.7314	0.8514	0.84	0.8457	0.8	0.8571
**Sensitivity**	0.7371	0.77143	0.7714	0.7714	0.72	0.8343	0.8229	0.8057	0.8228	0.8171
	**SVM Cubic 1**	**SVM Cubic 2**	**SVM Cubic 3**	**SVM Cubic 4**	**SVM Cubic 5**	**SVM Gaus 1**	**SVM Gaus 2**	**SVM Gaus 3**	**SVM Gaus 4**	**SVM Gaus 5**
**Accuracy**	0.80857	0.82	0.8257	0.81143	0.82	0.7643	0.7914	0.8029	0.8028	0.8114
**Specificity**	0.8571	0.8571	0.88	0.8457	0.8629	0.7486	0.7486	0.76	0.7714	0.76
**Sensitivity**	0.76	0.78286	0.7714	0.7771	0.7771	0.84	0.8343	0.8457	0.8343	0.8629

**Table 2:** Classifiers’ performance of each Best Food Source from each classifier.

With ABC										
	**Random 1**	**Random 2**	**Random 3**	**Random 4**	**Random 5**	**Tree Fine 1**	**Tree Fine 2**	**Tree Fine 3**	**Tree Fine 4**	**Tree Fine 5**
**Accuracy**	0.8362	0.8591	0.8667	0.8391	0.8543	0.74381	0.7114	0.7209	0.719	0.7229
**Specificity**	0.7981	0.8171	0.8229	0.8	0.8343	0.74	0.7143	0.7314	0.7486	0.72
**Sensitivity**	0.8743	0.901	0.9105	0.8781	0.8743	0.7467	0.7086	0.7105	0.6895	0.7257
	**Tree Med 1**	**Tree Med 2**	**Tree Med 3**	**Tree Med 4**	**Tree Med 5**	**Tree Coarse 1**	**Tree Coarse 2**	**Tree Coarse 3**	**Tree Coarse 4**	**Tree Coarse 5**
**Accuracy**	0.74	0.7343	0.7162	0.7191	0.7343	0.7238	0.7286	0.6905	0.6781	0.7057
**Specificity**	0.7486	0.7314	0.7371	0.7067	0.7257	0.6781	0.6495	0.6705	0.6133	0.6286
**Sensitivity**	0.7314	0.7371	0.6952	0.7314	0.7429	0.7695	0.8076	0.7105	0.7429	0.7829
	**kNN Fine 1**	**kNN Fine 2**	**kNN Fine 3**	**kNN Fine 4**	**kNN Fine 5**	**kNN Med 1**	**kNN Med 2**	**kNN Med 3**	**kNN Med 4**	**kNN Med 5**
**Accuracy**	0.8543	0.8986	0.86	0.8486	0.8486	0.8543	0.8571	0.88	0.8743	0.8629
**Specificity**	0.9314	0.92	0.9029	0.9371	0.9486	0.9714	0.9486	0.9314	0.92	0.9371
**Sensitivity**	0.7771	0.8171	0.8171	0.76	0.7486	0.7371	0.7657	0.8286	0.8286	0.7886
	**kNN Coarse 1**	**kNN Coarse 2**	**kNN Coarse 3**	**kNN Coarse 4**	**kNN Coarse 5**	**kNN Cosine 1**	**kNN Cosine 2**	**kNN Cosine 3**	**kNN Cosine 4**	**kNN Cosine 5**
**Accuracy**	0.88	0.8771	0.8657	0.8829	0.8914	0.8629	0.8486	0.8686	0.8371	0.8657
**Specificity**	0.9486	0.9086	0.9257	0.9543	0.9429	0.9371	0.92	0.9657	0.8914	0.9314
**Sensitivity**	0.8114	0.8457	0.8057	0.8114	0.84	0.7886	0.7771	0.7714	0.7829	0.8
	**SVM Linear 1**	**SVM Linear 2**	**SVM Linear 3**	**SVM Linear 4**	**SVM Linear 5**	**SVM Poly 1**	**SVM Poly 2**	**SVM Poly 3**	**SVM Poly 4**	**SVM Poly 5**
**Accuracy**	0.7686	0.7829	0.78	0.78	0.7486	0.8286	0.8057	0.7971	0.82	0.8171
**Specificity**	0.76	0.7829	0.7543	0.7829	0.72	0.8686	0.8057	0.8057	0.8571	0.8457
**Sensitivity**	0.7771	0.7829	0.8057	0.7771	0.7771	0.7886	0.8057	0.7886	0.84	0.7886
	**SVM Cubic 1**	**SVM Cubic 2**	**SVM Cubic 3**	**SVM Cubic 4**	**SVM Cubic 5**	**SVM Gaus 1**	**SVM Gaus 2**	**SVM Gaus 3**	**SVM Gaus 4**	**SVM Gaus 5**
**Accuracy**	0.8429	0.8514	0.8429	0.8229	0.8343	0.8171	0.8171	0.8314	0.8	0.8314
**Specificity**	0.8743	0.88	0.88	0.8457	0.8	0.7829	0.7771	0.7714	0.76	0.8114
**Sensitivity**	0.8114	0.8229	0.8057	0.8	0.8114	0.8514	0.8571	0.8914	0.84	0.8514

**Table 3:** The Best, Median, Mean, and SD of Accuracy for each classifier without using ABC.

WITHOUT ABC							
	4th Quantile	3rd Quantile	2nd Quantile	1st Quantile	0th Quantile		
Classifier	Best Acc	Quantile	Median	Quantile	Worst Acc	Mean	SD
**Random Forest**	0.791	0.78	0.777	0.762	0.76	0.7740	0.0130
**Decision Tree Fine**	0.7229	0.72	0.714	0.6914	0.6912	0.7079	0.0155
**Decision Tree Medium**	0.72	0.7171	0.6824	0.682	0.68	0.6963	0.0204
**Decision Tree Coarse**	0.7	0.69	0.68	0.66	0.6457	0.6751	0.0221
**kNN Fine**	0.8543	0.84	0.84	0.8314	0.8314	0.8394	0.0094
**kNN Medium**	0.8571	0.8543	0.8486	0.8429	0.8343	0.8474	0.0092
**kNN Coarse**	0.8657	0.8657	0.8543	0.8486	0.8429	0.8554	0.0102
**kNN Cosine**	0.7886	0.7829	0.78	0.7771	0.76	0.7777	0.0108
**SVM Linear**	0.7571	0.7543	0.7486	0.74	0.7257	0.7451	0.0127
**SVM Polynomial**	0.8371	0.8314	0.8257	0.82	0.8114	0.8251	0.0100
**SVM Cubic**	0.8257	0.82	0.82	0.81143	0.80857	0.8171	0.0070
**SVM Gaussian (RBF)**	0.8114	0.8029	0.8028	0.7914	0.7643	0.7946	0.0183

**Table 4:** The Best, Median, Mean, and SD of Accuracy of each classifier on each Best Food locations derived from ABC feature selection.

WITH ABC							
	4th Quantile	3rd Quantile	2nd Quantile	1st Quantile	0th Quantile		
Classifier	Best Acc	Quantile	Median	Quantile	Worst Acc	Mean	SD
**Random Forest**	0.8667	0.8591	0.8543	0.8391	0.8362	0.8511	0.0131
**Decision Tree Fine**	0.74381	0.7229	0.7209	0.719	0.7114	0.7236	0.0121
**Decision Tree Medium**	0.74	0.7343	0.7343	0.7191	0.7162	0.7288	0.0105
**Decision Tree Coarse**	0.7286	0.7238	0.7057	0.6905	0.6781	0.7053	0.0215
**kNN Fine**	0.8986	0.86	0.8543	0.8486	0.8486	0.8620	0.0210
**kNN Medium**	0.88	0.8743	0.8629	0.8571	0.8543	0.8657	0.0111
**kNN Coarse**	0.8914	0.8829	0.88	0.8771	0.8657	0.8794	0.0093
**kNN Cosine**	0.8686	0.8657	0.8629	0.8486	0.8371	0.8566	0.0133
**SVM Linear**	0.7829	0.78	0.78	0.7686	0.7486	0.7720	0.0142
**SVM Polynomial**	0.8371	0.8314	0.8257	0.82	0.8114	0.8251	0.0100
**SVM Cubic**	0.8514	0.8429	0.8429	0.8343	0.8229	0.8389	0.0108
**SVM Gaussian (RBF)**	0.8314	0.8314	0.8171	0.8171	0.8	0.8194	0.0130

**Table 5:** *t*-Test between using ABC and without ABC on the Accuracy value of each classifier.

		N	Mean	STD	DF	*t*	*p*-Value
**Random Forest**	With ABC	5.000	0.851	0.013	4.000	8.972	0.001
	Without ABC	5.000	0.774	0.013			*p* < 0.01
**Decision Tree Fine**	With ABC	5.000	0.724	0.012	4.000	2.124	0.051
	Without ABC	5.000	0.708	0.015			*p* < 0.10
**Decision Tree Medium**	With ABC	5.000	0.729	0.010	4.000	4.873	0.004
	Without ABC	5.000	0.696	0.020			*p* < 0.01
**Decision Tree Coarse**	With ABC	5.000	0.705	0.021	4.000	3.448	0.013
	Without ABC	5.000	0.675	0.022			*p* < 0.05
**kNN Fine**	With ABC	5.000	0.862	0.021	4.000	4.110	0.007
	Without ABC	5.000	0.839	0.009			*p* < 0.01
**kNN Medium**	With ABC	5.000	0.866	0.011	4.000	10.570	0.000
	Without ABC	5.000	0.847	0.009			*p* < 0.01
**kNN Coarse**	With ABC	5.000	0.879	0.009	4.000	12.491	0.000
	Without ABC	5.000	0.855	0.010			*p* < 0.01
**kNN Cosine**	With ABC	5.000	0.857	0.013	4.000	37.090	0.000
	Without ABC	5.000	0.778	0.011			*p* < 0.01
**SVM Linear**	With ABC	5.000	0.772	0.014	4.000	18.596	0.000
	Without ABC	5.000	0.745	0.013			*p* < 0.01
**SVM Polynomial**	With ABC	5.000	0.825	0.010	4.000	8.881	0.001
	Without ABC	5.000	0.814	0.012			*p* < 0.01
**SVM Cubic**	With ABC	5.000	0.839	0.011	4.000	11.259	0.001
	Without ABC	5.000	0.817	0.007			*p* < 0.01
**SVM Gaussian (RBF)**	With ABC	5.000	0.819	0.013	4.000	6.807	0.001
	Without ABC	5.000	0.795	0.018			*p* < 0.01

**Table 6:** The confusion matrix result from the non-train data.

Classifier		Accuracy	Sensitivity	Specificity
Decision Tree	Without ABC	0.667	0.778	0.556
	With ABC	0.833	1.000	0.667
Random Forest	Without ABC	0.944	1.000	0.889
	With ABC	1.000	1.000	1.000
kNN	Without ABC	0.944	0.889	1.000
	With ABC	0.944	0.889	1.000
SVM	Without ABC	0.944	0.889	1.000
	With ABC	0.944	0.889	1.000

## Data Availability

The dataset used in this study is available online at https://drive.google.com/drive/folders/1FjSqeCVtjl7hZvMj1CwphgWJGx8XoCAr?usp=sharing (accessed on 9 April 2021).
